# The Hereditary Angioedema Frailty and Inflammation Score (HAE-FIS): A Preliminary Study on the Inter-Attack Chronic Burden

**DOI:** 10.3390/jcm15093417

**Published:** 2026-04-29

**Authors:** Ayşe Melike Gerek, Fatih Çölkesen, Şevket Arslan

**Affiliations:** 1 Department of Physical Medicine and Rehabilitation, Konya Numune Hospital, Ferhuniye Mah, Hastane Cd. No. 22, Selçuklu, 42060 Konya, Türkiye; 2 Department of Internal Medicine, Division of Clinical Immunology and Allergy, Faculty of Medicine, Necmettin Erbakan University, Hocacihan Mahallesi, Sultan Abdülhamid Han Caddesi No. 3, Selçuklu, 42280 Konya, Türkiye; fcolkesen@erbakan.edu.tr (F.Ç.); sevketarslan@erbakan.edu.tr (Ş.A.)

**Keywords:** C1 inhibitor deficiency, chronic inflammation, frailty, hereditary angioedema, rehabilitation

## Abstract

**Background/Objectives:** Current management of Hereditary Angioedema (HAE) predominantly focuses on acute attack control and prophylaxis. However, the cumulative “inter-attack” burden driven by chronic low-grade inflammation and its impact on physical frailty remain under-investigated. This preliminary, proof-of-concept study proposes a novel composite score, the “Hereditary Angioedema Frailty and Inflammation Score” (HAE-FIS), to quantify this inter-attack frailty-like inflammatory burden. **Methods:** In this single-center retrospective study, 46 patients with C1-inhibitor deficiency were evaluated. The HAE-FIS (range 0–5) was constructed using five routinely available biomarkers reflecting inflammation and nutritional status: C-reactive protein (CRP), albumin, hemoglobin, body mass index (BMI), and inter-attack symptom burden. Patients were categorized based on annual attack frequency (Infrequent ≤ 6 vs. Frequent > 6 attacks/year). **Results:** The median diagnostic delay was 12.0 years. Patients with frequent attacks had significantly higher median HAE-FIS scores compared to the infrequent group (*p* = 0.008). Component analysis revealed that ‘High CRP’ (46.4% vs. 16.7%; *p* = 0.039) and ‘Low BMI’ (25.0% vs. 0.0%; *p* = 0.032) were significantly more prevalent in patients with frequent attacks. Notably, low BMI was observed exclusively in the frequent attack group, suggesting a specific phenotype at risk for sarcopenia. While multivariable logistic regression was constrained by the sample size, the annual attack count emerged as the strongest predictor for a high HAE-FIS score (OR: 1.049; *p* = 0.070). **Conclusions:** High attack frequency in HAE is associated with a measurable cumulative systemic burden characterized by inflammation and nutritional risk. These findings support the development of an “HAE Rehabilitation” framework integrating functional preservation into long-term management.

## 1. Introduction

Hereditary Angioedema (HAE) is a rare genetic disorder characterized by recurrent and unpredictable bradykinin-mediated angioedema attacks resulting from C1 inhibitor deficiency or dysfunction [1]. In clinical practice and scientific literature, HAE management has largely focused on the treatment of acute attacks and long-term prophylaxis strategies. However, this approach risks overlooking subclinical pathophysiological processes persisting during the “inter-attack” period. Consequently, the cumulative effects of these processes on the patient’s general physical health and functional capacity may be neglected.

Recent evidence indicates that HAE is not merely an episodic disease. Endothelial dysfunction and persistent, low-grade systemic inflammation continue even during asymptomatic periods [2,3]. This chronic inflammatory background falls within the core scope of Physical Medicine and Rehabilitation (PM&R). It can trigger cytokine-mediated processes that accelerate muscle protein catabolism, leading to clinical manifestations such as fatigue, anorexia, and anemia [4]. Consequently, these pathophysiological alterations suggest that HAE patients may be predisposed to a ‘frailty-like’ syndrome. This syndrome is characterized by loss of muscle mass (sarcopenia), nutritional impairment, and reduced physiological reserve over time.

Literature in PM&R and geriatrics has long emphasized that low-grade inflammation is a fundamental risk factor for sarcopenia and functional capacity loss in chronic inflammatory diseases [5]. However, this important hypothesis represents an area that has not yet been systematically investigated in the HAE literature.

To address this gap, this study proposes a new composite score aimed at evaluating this chronic burden during the inter-attack period. This tool is termed the “Hereditary Angioedema Frailty and Inflammation Score” (HAE-FIS). The score combines five well-established parameters proven to be associated with frailty, malnutrition, and poor prognosis: high C-reactive protein (CRP), low serum albumin, anemia, low body mass index (BMI), and symptom burden. The fundamental principle of this score is to use simple biomarkers accessible in routine clinical practice rather than complex laboratory indicators. The aim is to provide clinicians with a cost-effective tool to identify which patients might benefit from proactive PM&R assessment and personalized rehabilitation.

In this context, the primary aim of this exploratory study is to investigate the relationship between the newly proposed HAE-FIS score and attack frequency, a key indicator of disease activity. Our hypothesis is that patients with frequent attacks demonstrate a greater cumulative inflammatory burden and consequently exhibit significantly higher HAE-FIS scores compared to patients with infrequent attacks. Secondarily, we investigated whether a difference existed in this score between HAE Type 1 and Type 2 groups. Thus, this study represents an exploratory but necessary step toward a clinical approach focusing on preserving physical function in addition to attack control.

## 2. Materials and Methods

### 2.1. Study Design

This study was designed as a single-center, cross-sectional, retrospective observational study based on medical record review. The study was conducted at the Necmettin Erbakan University Meram Faculty of Medicine Hospital, Department of Allergy and Immunology, covering the period from 1 January 2020 to 31 December 2024.

### 2.2. Ethical Approval

The study protocol was approved by the Necmettin Erbakan University Ethics Committee for Non-Pharmaceutical and Non-Medical Device Research (2025/6064). All procedures were conducted in accordance with the ethical standards of the institutional research committee and with the 1964 Helsinki Declaration and its later amendments or comparable ethical standards. Due to the retrospective nature of the study, the requirement for informed consent was waived by the ethics committee.

### 2.3. Study Population and Sample

The study population consisted of patients followed for a diagnosis of Hereditary Angioedema (HAE) Type 1 or Type 2 due to C1 inhibitor deficiency at the Necmettin Erbakan University Faculty of Medicine Hospital, Department of Allergy and Immunology. All data were retrieved retrospectively from the Hospital Information Management System (HIMS) and electronic patient files.

**Figure 1:**
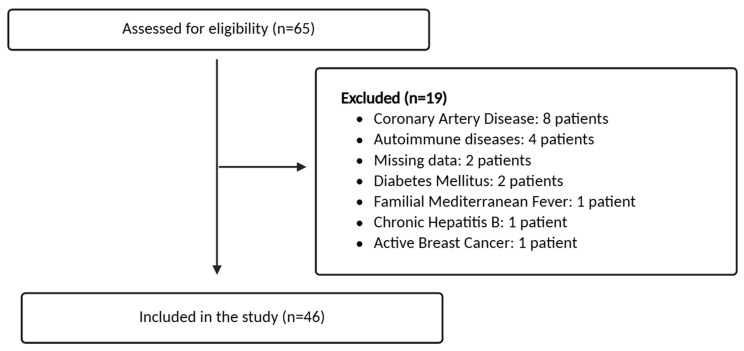
Flowchart illustrating the patient selection process and reasons for exclusion.

A total of 65 patients with a confirmed HAE diagnosis were initially screened. Following the application of inclusion and exclusion criteria, 19 patients were excluded, and 46 patients were included in the final analysis. The patient selection process is summarized in Figure 1.

Inclusion Criteria:

- Confirmed diagnosis of HAE Type 1 or Type 2 according to international guidelines.
- Age 18 years or older.
- Regular follow-up at the study center for at least 1 year.

Exclusion Criteria:

- Age under 18 years.
- Missing data for any of the laboratory or anthropometric parameters required for analysis.
- Presence of any known comorbid condition with potential to independently affect inflammatory or nutritional biomarkers, including cardiovascular disease (e.g., coronary artery disease), autoimmune diseases, active malignancy, diabetes mellitus, or chronic infectious/inflammatory conditions (e.g., Familial Mediterranean Fever, Chronic Hepatitis B).

### 2.4. Variables and Definitions

To ensure that laboratory parameters reflected the chronic inter-attack inflammatory status rather than acute-phase reactivity, only measurements obtained at a routine follow-up visit at least 14 days after the complete resolution of the most recent angioedema attack were included. This washout interval is supported by the established kinetics of both biomarkers: CRP, a positive acute-phase reactant with a plasma half-life of approximately 19 h, normalizes within 1–2 weeks following resolution of acute inflammation; and serum albumin, a negative acute-phase reactant whose hepatic synthesis is suppressed during inflammation and recovers toward baseline within 2–4 weeks after inflammatory resolution [6,7,8]. This approach is further consistent with the methodology employed in prior HAE inter-attack biomarker studies [9].

#### 2.4.1. HAE Frailty and Inflammation Score (HAE-FIS)

In this study, a composite score ranging from 0 to 5 was developed to evaluate the burden of frailty and inflammation in HAE patients. The five components were selected to reflect distinct pathophysiological aspects of the chronic burden: CRP and albumin as markers of systemic inflammation and nutritional decline; BMI as a surrogate for catabolic muscle loss; hemoglobin for chronic disease anemia; and neuropathic medication use as an indicator of the chronic pain burden and central sensitization that have been reported in HAE patients. Other potential inflammatory markers (e.g., IL-6, ESR) were not included because they were inconsistently measured across the cohort, whereas the selected items were universally available in routine clinical records. One point was assigned for the presence of each of the following five criteria:

1. **High Inflammation:** C-reactive protein (CRP) > 5 mg/L [10].
2. **Anemia:** Hemoglobin < 12 g/dL (Female) or <13 g/dL (Male) [11].
3. **Low Body Mass Index (BMI):** BMI < 21 kg/m^2^ [12].
4. **Low Albumin:** Serum Albumin < 3.8 g/dL [13].
5. **Symptom Burden:** Regular prescription of a drug used in the treatment of neuropathic pain/symptoms, such as opioids, gabapentinoids, or tricyclic antidepressants, for a minimum of three months within the preceding year [14]. Chronic post-attack neuropathic pain syndromes have been described in HAE patients, particularly following recurrent abdominal attacks, supporting the rationale for including these medications as an indicator of persistent symptom burden. Crucially, prescriptions strictly for on-demand therapy of acute angioedema attacks were excluded to prevent confounding with attack frequency.

#### 2.4.2. Clinical Groupings

- **Attack Frequency:** Patients were divided into two groups according to their annual attack frequency: Infrequent Attack (≤6 attacks/year) and Frequent Attack (>6 attacks/year).
- **HAE-FIS Group:** Patients were divided into two main groups according to the composite score to facilitate subsequent regression analysis and to increase the clinical interpretability of the score: Low FIS Group (Score = 0) and High FIS Group (Score ≥ 1). There were three fundamental rationales for this binary grouping:

1. **Statistical Requirement:** This grouping served as a statistical prerequisite to ensure the dependent variable was binary for the planned multivariable logistic regression analysis to determine independent risk factors predicting a High FIS score.
2. **Clinical Interpretability:** Simplifying the score distribution between 0 and 4 into “no risk/low risk” (Score = 0) and “increased risk” (Score ≥ 1) aimed to facilitate the understanding and potential use of the score by clinicians.
3. **Objective Cut-Off Point:** The selection of score ‘1’ as the cut-off point between groups was based on the median FIS score of the entire study cohort (N = 46) rather than an arbitrary decision. This aimed to ensure a data-driven and objective grouping method.

### 2.5. Statistical Analysis

All statistical analyses were performed using the IBM SPSS Statistics for Windows, Version 22.0 (IBM Corp., Armonk, NY, USA) software program. The conformity of continuous variables to normal distribution was evaluated using the Shapiro–Wilk test.

Descriptive statistics were presented as Mean ± Standard Deviation (SD) for normally distributed variables, Median [Interquartile Range (IQR) or Minimum-Maximum] for non-normally distributed variables, and number (*n*) and percentage (%) for categorical variables.

For comparisons between groups, Student’s *t*-test was used for normally distributed continuous variables, and the Mann–Whitney U test was used for non-normally distributed variables. The Chi-Square test or Fisher’s Exact Test was used for categorical variables, as appropriate based on expected cell counts.

Variables found to have *p* < 0.10 in univariate analysis were included in the multivariable binary logistic regression model using the ‘Enter’ method to identify independent predictors of a high FIS score. Given the rarity of the disease and the resulting small sample size, the ‘events per variable’ (EPV) ratio was lower than traditionally recommended. Therefore, the multivariable analysis was conducted and interpreted as an exploratory hypothesis-generating model rather than a confirmatory analysis, acknowledging the potential risk of overfitting and the need for validation in larger cohorts. Model fit was evaluated using the Hosmer-Lemeshow test. A two-sided *p*-value of <0.05 was considered statistically significant for all analyses.

To address the potential for incorporation bias arising from CRP being both a component of the HAE-FIS and an independent variable in subsequent analyses, a pre-specified sensitivity analysis was conducted. In this analysis, CRP was excluded from the composite score, yielding a 4-component HAE-FIS (range 0–4). All primary analyses were repeated with this modified score.

## 3. Results

### 3.1. Demographic and Clinical Characteristics of the Participants

A total of 46 patients followed up with a diagnosis of Hereditary Angioedema (HAE) due to C1 inhibitor deficiency were included in the study. The mean age of the participants was 36.7 ± 11.3 years, and 52.2% (*n* = 24) were female. Of the participants, 56.5% (*n* = 26) had HAE Type 1, while 43.5% (*n* = 20) had HAE Type 2.

A family history was present in most patients (78.3%, *n* = 36). The mean age at onset of first symptoms was 12.9 ± 12.0 years, while the mean age at diagnosis was 26.3 ± 12.1 years, indicating a median diagnostic delay of 12.0 years. Regarding attack frequency, 60.9% (*n* = 28) of the patients were in the “frequent attack” group, experiencing more than 6 attacks per year. The most frequently reported first symptom location was the abdominal region (54.3%, *n* = 25). Detailed demographic, clinical, and laboratory characteristics of the study group are summarized in Table 1.

### 3.2. Evaluation of HAE-FIS Score and Components According to Attack Frequency

To evaluate the association between attack frequency and HAE-FIS score, consistent with one of the primary study hypotheses, patients were divided into two groups: infrequent (≤6 attacks/year) and frequent (>6 attacks/year).

Statistically significant differences were observed between the two groups in terms of both the total HAE-FIS score and the distribution of the score. The median FIS score of the frequent attack group was significantly higher than that of the infrequent attack group (*p* = 0.008). This difference was primarily attributable to the observation that high FIS scores (score ≥ 2) were observed exclusively in the frequent attack group (*p* = 0.049).

Component-level analysis revealed that the prevalence of ‘High CRP’ (46.4% vs. 16.7%; *p* = 0.039) and ‘Low BMI’ (25.0% vs. 0.0%; *p* = 0.032) was significantly higher in the frequent attack group. Notably, low BMI was observed exclusively in the frequent attack group (25.0%), suggesting a specific phenotype at risk for cachexia and sarcopenia. All these findings are summarized in Table 2.

### 3.3. Factors Associated with High HAE-FIS Score

To evaluate the main outcome of the study, patients were divided into two groups according to their HAE-FIS scores: Low FIS Group (Score = 0, *n* = 20) and High FIS Group (Score ≥ 1, *n* = 26). Demographic, clinical, and laboratory characteristics of these two groups were compared.

Patients with a high FIS score had a significantly higher median annual attack count (32.0 vs. 5.0; *p* = 0.004) and higher median CRP levels (3.0 mg/L vs. 2.3 mg/L; *p* = 0.002) compared to patients with a low score. Additionally, a high FIS score was found to be associated with an older age of first symptom onset (median 12.0 vs. 8.0 years; *p* = 0.045) and an older age at diagnosis (mean 28.0 vs. 24.0 years; *p* = 0.041).

No statistically significant difference was found between the groups in terms of other demographic, clinical, and laboratory characteristics. Findings are presented in detail in Table 3.

### 3.4. Independent Risk Factors for High HAE-FIS Score (Multivariable Logistic Regression Analysis)

The multivariable logistic regression model, constructed to identify independent predictors of a high FIS score (score ≥ 1), demonstrated a good fit with the data (Hosmer-Lemeshow test, *p* = 0.856). The model was statistically significant overall (χ^2^(6) = 23.770, *p* = 0.001) and accounted for approximately 54.1% of the pseudo-variance in the FIS score (Nagelkerke R^2^ = 0.541). The multivariable model should be interpreted cautiously due to the limited events-per-variable (EPV) ratio inherent to rare disease cohorts.

However, none of the variables included in the model emerged as independent predictors of a high FIS score after adjusting for other factors (all *p*-values > 0.05). In the multivariable analysis, two variables approached statistical significance: Age (*p* = 0.062) and Annual Attack Count (*p* = 0.070). The Annual Attack Count demonstrated an Odds Ratio (OR) of 1.049, indicating that each additional attack per year was associated with an approximately 4.9% increase in the likelihood of belonging to the high FIS group. Conversely, Age showed an OR of 0.914, suggesting that younger age was associated with a trend toward higher HAE-FIS scores. Detailed results of the model are presented in Table 4.

### 3.5. Sensitivity Analysis (CRP-Excluded HAE-FIS)

When CRP was excluded from the composite score, the 4-component HAE-FIS (range 0–4) continued to demonstrate a statistically significant association with attack frequency. The median score remained significantly higher in the frequent attack group compared to the infrequent group (1.0 [IQR 0–2] vs. 0.0 [IQR 0–0]; *p* = 0.016). Furthermore, a High 4-component FIS score (≥1) was associated with a markedly higher annual attack count (Median 60.0 vs. 6.0; *p* = 0.001). These findings indicate that the primary associations observed in the main analysis are robust and not an artifact of incorporating CRP into both the score and the predictor analysis.

To further address the potential for incorporation bias, the multivariable logistic regression model was repeated using the 4-component HAE-FIS (≥1) as the dependent variable, with CRP removed from both the composite score and the set of covariates. In this sensitivity model, the annual attack count emerged as a statistically significant independent predictor of a high 4-component FIS score (OR: 1.042; 95% CI: 1.007–1.079; *p* = 0.019). Age (OR: 0.883; *p* = 0.031) and gender (OR: 0.109; *p* = 0.025) also reached statistical significance. The model demonstrated adequate fit (Hosmer-Lemeshow test, *p* = 0.237) and was significant overall (*p* < 0.001; Nagelkerke R^2^ = 0.392). These findings confirm that the association between disease activity and the frailty-inflammation burden is robust and independent of CRP’s dual role in the primary analysis.

## 4. Discussion

This study evaluates the chronic inflammatory burden and consequent physical frailty in patients with HAE due to C1 inhibitor deficiency, assessed through the clinical associations of the newly developed composite score, HAE-FIS. This work introduces the concepts of functional loss and frailty associated with chronic diseases, which are central interests of PM&R, into the field of HAE. It provides preliminary data supporting a shift from a purely attack-focused approach toward a more holistic model that addresses the chronic and multidimensional burden of the disease [15,16].

The primary finding of our study is that the annual attack frequency, an indicator of disease activity, is strongly associated with the proposed HAE-FIS score. According to our results, the median HAE-FIS scores of patients with frequent attacks (>6 attacks/year) were significantly higher than those of patients with infrequent attacks. This finding supports our hypothesis that recurrent attacks create a cumulative and measurable systemic burden that may lead to functional loss over time, extending their impact well beyond the acute immunological episode itself. Importantly, this observation is not unique to C1 inhibitor deficiency-related HAE; analogous low-grade inflammation patterns have been reported in other angioedema and vascular permeability disorders involving complement system activation and bradykinin-mediated mechanisms, supporting the biological plausibility of our findings [17,18].

In our study, the difference in HAE-FIS scores between the frequent and infrequent attack groups was primarily driven by two components: ‘High CRP’ and ‘Low BMI’. The presence of elevated CRP in nearly half (46.4%) of the patients in the frequent attack group is consistent with persistent low-grade inflammation in this subgroup. Chronic inflammation is known to increase muscle protein catabolism, leading to fatigue and pain—a key mechanism underlying the development of ‘frailty’ and ‘sarcopenia’ in PM&R practice [9,19,20]. Similarly, the observation of low BMI prevalence exclusively in the frequent attack group (25.0%) highlights the risk of cachexia and sarcopenia resulting from this chronic catabolic process. It is well-established that increased cytokine levels in chronic inflammatory diseases promote muscle protein degradation, leading to reduced functional capacity [21,22]. This relationship demonstrates that inflammation-induced sarcopenia and frailty are not limited to elderly populations but also involve similar mechanisms in chronic inflammatory diseases [2,23]. Taken together, these findings suggest that high disease activity may initiate or perpetuate a catabolic cycle that threatens the musculoskeletal integrity and overall functional capacity, supporting the potential value of proactive rehabilitative assessment in this population. In this study, the term ‘frailty-like burden’ is used to describe the chronic functional vulnerability arising from inflammation-driven catabolic processes, distinct from geriatric frailty but mechanistically related to sarcopenia and decreased physiological reserve. Furthermore, although not directly measured in this study, the potential role of ‘kinesiophobia’ (fear of movement) warrants consideration as an additional contributing factor, albeit with appropriate caution. Previous studies have documented elevated anxiety and movement-avoidance behaviors in patients with chronic diseases [24,25]. These behaviors may indirectly contribute to physical deconditioning, operating independently of inflammation. Patients with frequent attacks may avoid physical activity to prevent trauma-induced angioedema, creating a vicious cycle of deconditioning and muscle atrophy independent of inflammation.

One of our initial hypotheses was that HAE Type 2 patients would have higher mean HAE-FIS scores than Type 1 patients, based on the assumption of a greater systemic inflammatory burden. However, our findings revealed no statistically significant difference in HAE-FIS scores between HAE Type 1 and Type 2 patients. From a PM&R perspective, this “negative” finding is nonetheless clinically meaningful. It suggests that functional impairment, muscle loss, and rehabilitation needs in HAE patients are more dominantly associated with clinical parameters reflecting phenotypic severity, such as attack frequency, rather than the underlying genetic defect type (low protein level vs. functional impairment). These findings suggest that eligibility for PM&R assessment and personalized rehabilitation may be more appropriately guided by clinical disease activity—specifically attack frequency and the resulting systemic burden—than by the underlying molecular mechanism of C1 inhibitor deficiency [26,27]. Nevertheless, the possibility that our study’s sample size (Type 1, *n* = 26; Type 2, *n* = 20) may have been insufficient in terms of statistical power to detect a potential small difference between the two groups should be considered.

An unexpected finding of our study was the association of a high FIS score with an older age of first symptom onset and an older age at diagnosis. This contradicts the clinical expectation that mild disease would be diagnosed later. The underlying reasons for this paradoxical relationship may be complex. One possible explanation is that late-onset HAE may represent a different biological phenotype leading to greater inflammatory burden accumulation over time; indeed, the literature data suggest that the age of symptom onset may influence the clinical phenotype [28]. Alternatively, given that this finding is close to the significance threshold, it may represent a chance finding attributable to multiple comparisons (Type I error), which should also be considered. Further prospective studies with larger cohorts are needed to clarify this issue.

Multivariable logistic regression analysis conducted to identify independent predictors of a high HAE-FIS score showed that the model was significant overall (Nagelkerke R^2^ = 0.54) and explained more than half of the score variance. This finding is consistent with the assumption that HAE-FIS components collectively capture the frailty-like and inflammatory burden in this population. Although our multivariable logistic regression model explained a significant portion of the variance (Nagelkerke R^2^ = 0.54), no single variable emerged as a statistically significant independent predictor. This is likely attributable to the limited sample size inherent to rare disease cohorts, which may have led to a Type II error (insufficient statistical power). However, the annual attack count showed a strong trend approaching significance (*p* = 0.070, OR: 1.049). Although younger age also exhibited a borderline trend (*p* = 0.062), likely reflecting early-onset phenotypic severity, the association with attack count clinically reinforces the link between disease activity and the cumulative inflammatory burden. From a PM&R perspective, this suggests that frailty-like vulnerability in HAE is unlikely to be fully addressed by a single parameter (e.g., only reducing the number of attacks) and may be better approached within a biopsychosocial model.

This complex interaction highlights the importance of an interdisciplinary approach, emphasizing the Immunologist’s role in controlling disease activity and the PM&R specialist’s role in managing the functional consequences (muscle strength loss, fatigue, pain) caused by this activity. Within this holistic model, the emergence of the annual attack count as the strongest candidate predictor (*p* = 0.070) indicates that immunological control is a fundamental prerequisite for the success of rehabilitation interventions. Therefore, although our analysis did not identify a single independent predictor, our findings support an interdisciplinary management model and corroborate the view that high attack frequency is a key dynamic factor potentially triggering rehabilitation needs [29,30].

The findings of this study should be evaluated within its methodological framework. A notable contribution of this study is the development of a pragmatic tool for assessing the chronic inter-attack burden in HAE—the HAE-FIS—comprising parameters universally available in routine clinical settings. This approach proposes a holistic perspective in disease management by bridging Immunology and Rehabilitation Medicine. Furthermore, the careful exclusion of other chronic diseases, such as rheumatoid arthritis or uncontrolled diabetes, which could affect the inflammation and frailty score, allows the observed findings to be more specifically associated with HAE.

However, the study has significant limitations that warrant careful interpretation. First, with respect to study design, the retrospective and cross-sectional nature precludes establishing a causal relationship between frequent attacks and a high HAE-FIS score, indicating only an association. The single-center design limits the generalizability of the results, while the small sample size (N = 46), inherent to rare disease research, may have reduced statistical power in multivariable analyses, increasing the risk of Type II error. Additionally, biomarker values represented a single point, limiting the longitudinal interpretation of inflammatory fluctuations.

Second, several limitations relate to the individual components of the HAE-FIS. A potential concern is the incorporation of CRP as both a score component and an analytical variable, which may introduce circularity. However, sensitivity analyses excluding CRP from both the composite score and the multivariable regression model yielded consistent results, suggesting that this dual role did not materially affect the primary conclusions. BMI was used as a surrogate marker for nutritional status and body composition; however, it is an imprecise measure of catabolic muscle loss, as reductions in BMI may reflect loss of fat mass, fluid shifts, or dehydration rather than sarcopenia specifically. This limitation is particularly relevant in HAE, where recurrent angioedema episodes may transiently alter fluid distribution and body weight. Furthermore, BMI cannot distinguish sarcopenic obesity—a condition where muscle loss coexists with preserved or elevated fat mass—which is a clinically relevant phenotype in chronic inflammatory diseases. Similarly, the operationalization of symptom burden through medication prescription records represents a pragmatic but imperfect proxy, potentially subject to local prescribing variation and unable to fully capture patient-reported functional impairment.

Third, the absence of direct functional assessments—such as handgrip strength, gait speed, or validated patient-reported outcome measures—limits the clinical interpretability of the frailty-like burden described in this study. Without confirmation that laboratory-derived risk translates into measurable functional impairment, the clinical relevance of the HAE-FIS as a standalone screening tool remains preliminary. Future prospective studies should incorporate direct body composition measurements (e.g., DEXA or bioimpedance analysis), validated functional performance tests, and patient-reported outcome measures to more precisely characterize the musculoskeletal and symptomatic burden in HAE.

Despite its limitations, our findings offer significant implications for the clinical management of patients with HAE and challenge traditional treatment paradigms. Based on these preliminary findings, we hypothesize that HAE management could evolve toward a dual-track care model that ensures immunological attack control while simultaneously monitoring and optimizing the patient’s functional status. This dual-track care model suggests a system where one track targets immunological activity, while the other focuses on muscle strength, range of motion, and quality of life. In this model, the HAE-FIS score warrants further investigation as a potential screening tool for identifying rehabilitation needs. A high score (e.g., ≥1 in our cohort) may serve as a practical screening flag—not a diagnostic instrument—to identify patients who may benefit from formal PM&R consultation, including direct functional assessment. This consultation should include a comprehensive functional assessment encompassing sarcopenia analysis (e.g., handgrip strength, gait speed), evaluation of chronic pain and fatigue, and nutritional screening. These measurements enable a biopsychosocial evaluation of the patient’s functional status in accordance with the World Health Organization’s ICF (International Classification of Functioning, Disability and Health) framework. Based on this assessment, the PM&R specialist, in collaboration with the immunologist, can design personalized interventions such as targeted exercise programs (e.g., progressive resistance exercises to combat sarcopenia), pain management strategies, and nutritional support.

This study also highlights an emerging research area that may be termed “HAE Rehabilitation.” Future research should prioritize the external validation of the proposed HAE-FIS score in larger, multicenter, prospective cohorts with adequate statistical power. Such studies should rigorously assess the score’s construct validity, internal consistency (e.g., Cronbach’s alpha), and determine optimal cut-off values using ROC curve analyses against established frailty instruments. In addition, future work should integrate not only physical performance indicators but also quality of life measures (e.g., EQ-5D, HAE-QoL) and neuromuscular endurance tests. Beyond this, studies are needed to clarify the specific phenotype of functional impairment in HAE (e.g., whether the primary driver is sarcopenia, deconditioning related to fear of movement, or chronic pain syndromes). The most critical next step will be intervention studies. Randomized controlled trials evaluating the effectiveness of targeted rehabilitation programs on objective functional outcomes (e.g., 6 min walk test, quality of life scales such as SF-36) and on the HAE-FIS score itself would help to establish the role of this interdisciplinary approach within evidence-based management.

## 5. Conclusions

This exploratory study demonstrates that the HAE-FIS score is significantly associated with disease activity in patients with Hereditary Angioedema, indicating that frequent attacks contribute to a measurable cumulative systemic burden that extends beyond the acute episode itself. These preliminary findings suggest that clinical management of HAE may benefit from an interdisciplinary approach that incorporates immunological treatment with rehabilitation strategies aimed at preserving functional capacity, pending validation in larger prospective cohorts. Collectively, these preliminary findings provide evidence that functional preservation represents a clinically relevant therapeutic target alongside immunological control in the long-term management of hereditary angioedema, a hypothesis that warrants prospective validation.

## Figures and Tables

**Table 1:** Demographic, clinical, and laboratory characteristics of the study group (N = 46).

Characteristic	Value
Demographic Data	
Age (years, Mean ± SD)	36.7 ± 11.3
Gender, Female, *n* (%)	24 (52.2%)
Clinical Characteristics	
HAE Type, Type 1, *n* (%)	26 (56.5%)
Family History, Present, *n* (%)	36 (78.3%)
Age at First Symptom (years, Mean ± SD)	12.9 ± 12.0
Age at Diagnosis (years, Mean ± SD)	26.3 ± 12.1
Diagnostic Delay (years, Median [Q1–Q3])	12.0 [2.8–22.0]
Annual Attack Frequency, Median [Q1–Q3]	24.0 [5.0–45.0]
Frequent Attack Group (>6 attacks/year), *n* (%)	28 (60.9%)
Laboratory and Anthropometric Findings	
Hemoglobin (g/dL, Mean ± SD)	14.2 ± 1.7
Albumin (g/dL, Mean ± SD)	4.56 ± 0.4
CRP (mg/L, Median [Q1–Q3])	2.7 [1.6–5.6]
Creatinine (mg/dL, Median [Q1–Q3])	0.75 [0.7–0.9]
AST (U/L, Median [Q1–Q3])	15.5 [13.1–19.9]
ALT (U/L, Median [Q1–Q3])	15.7 [11.9–22.3]
C4 (mg/dL, Median [Q1–Q3])	6.8 [4.8–9.3]
C1 Inhibitor Level (%, Median [Q1–Q3])	11.0 [5.8–34.3]
C1 Inhibitor Function (%, Median [Q1–Q3])	10.5 [7.0–17.6]
Body Mass Index (kg/m^2^, Mean ± SD)	28.1 ± 6.2

Notes: SD: Standard Deviation, Q1: 25th percentile, Q3: 75th percentile, HAE: Hereditary Angioedema, CRP: C-reactive protein, AST: Aspartate aminotransferase, ALT: Alanine aminotransferase.

**Table 2:** Comparison of HAE-FIS score, distribution, and components according to attack frequency groups.

Characteristic	Infrequent Attack Group (N = 18)	Frequent Attack Group (N = 28)	*p*-Value
Total HAE-FIS Score (Median [Min–Max])	0.0 [0–1]	1.0 [0–4]	0.008 ^a^
**HAE-FIS Score Distribution, *n* (%)**			0.049 ^b^
Score = 0	11 (61.1%)	9 (32.1%)	
Score = 1	7 (38.9%)	8 (28.6%)	
Score ≥ 2	0 (0.0%)	11 (39.3%)	
**HAE-FIS Components, *n* (%)**			
High CRP (>5 mg/L)	3 (16.7%)	13 (46.4%)	0.039 ^b^
Low BMI (<21 kg/m^2^)	0 (0.0%)	7 (25.0%)	0.032 ^c^
Symptom Burden (Medication Use)	1 (5.6%)	8 (28.6%)	0.055 ^b^
Low Albumin (<3.8 g/dL)	0 (0.0%)	3 (10.7%)	0.151 ^b^
Anemia	3 (16.7%)	6 (21.4%)	0.691 ^b^

Notes: ^a^ Mann–Whitney U test. ^b^ Chi-Square test. ^c^ Fisher’s Exact Test.

**Table 3:** Comparative analysis of groups with low and high HAE-FIS scores.

Characteristic	Low FIS Group (Score = 0, N = 20)	High FIS Group (Score ≥ 1, N = 26)	*p*-Value
Disease Activity			
Annual Attack Count, Median [Q1–Q3]	5.0 [2.8–12.0]	32.0 [13.8–50.0]	0.004
Frequent Attack Group (>6 attacks/year), *n* (%)	9 (45.0%)	19 (73.1%)	0.053
Demographic and Clinical Data			
Age (years, Mean ± SD)	36.2 ± 12.2	36.7 ± 10.7	0.944
Gender, Female, *n* (%)	9 (45.0%)	15 (57.7%)	0.393
HAE Type, Type 1, *n* (%)	12 (60.0%)	14 (53.8%)	0.676
Age at First Symptom (years, Median [Q1–Q3])	8.0 [4.0–16.0]	12.0 [6.0–18.8]	0.045
Age at Diagnosis (years, Mean ± SD)	24.0 ± 12.7	28.0 ± 11.6	0.041
Diagnostic Delay (years, Median, IQR 25–75)	15.0 [7.0–22.0]	11.0 [0.8–22.0]	0.850
Attack History			
Mucocutaneous Attack, Present, *n* (%)	19 (95.0%)	24 (92.3%)	0.714
Gastrointestinal Attack, Present, *n* (%)	12 (60.0%)	21 (80.8%)	0.121
Laryngeal Attack, Present, *n* (%)	4 (20.0%)	9 (34.6%)	0.275
Laboratory Findings			
C4 (mg/dL, Median [Q1–Q3])	8.6 [5.0–10.5]	6.5 [4.4–8.8]	0.096
C1 Inhibitor Level (%, Median [Q1–Q3])	12.0 [4.3–36.3]	11.0 [6.8–30.5]	0.587
C1 Inhibitor Function (%, Median [Q1–Q3])	10.5 [6.8–14.3]	10.5 [7.5–19.3]	0.587

Notes: Continuous variables were compared using the Mann–Whitney U test, and categorical variables using the Chi-Square test. Data are presented as Mean ± Standard Deviation (SD) or Median [Q1–Q3].

**Table 4:** Results of multivariable logistic regression analysis for High HAE-FIS score (Score ≥ 1).

Variable	*p*-Value	OR (Exp(B))	95% Confidence Interval (CI)
Annual Attack Count	0.070	1.049	0.996–1.105
CRP (mg/L)	0.129	1.447	0.898–2.333
Age at First Symptom (years)	0.695	1.018	0.931–1.113
C4 (mg/dL)	0.448	1.075	0.892–1.294
Age (years)	0.062	0.914	0.832–1.004
Gender (Male vs. Female)	0.569	1.749	0.255–11.990

Notes: OR: Odds Ratio, CI: Confidence Interval. Overall model significance (Omnibus Test): χ^2^(6) = 23.770, *p* = 0.001. Nagelkerke R^2^ = 0.541. Model fit (Hosmer-Lemeshow Test): *p* = 0.856.

## Data Availability

The data presented in this study are available on request from the corresponding author due to privacy restrictions related to patient information.

## References

[1] Miyata T., Horiuchi T. (2023). Biochemistry, Molecular Genetics, and Clinical Aspects of Hereditary Angioedema with and without C1 Inhibitor Deficiency. Allergol. Int..

[2] Cruz-Jentoft A.J., Bahat G., Bauer J., Boirie Y., Bruyère O., Cederholm T., Cooper C., Landi F., Rolland Y., Sayer A.A. (2019). Sarcopenia: Revised European Consensus on Definition and Diagnosis. Age Ageing.

[3] Loffredo S., Ferrara A., Bova M., Borriello F., Suffritti C., Veszeli N., Yoshida T., Zanichelli A., Marcos C.M., Cicardi M. (2018). Secreted Phospholipases A2 in Hereditary Angioedema with C1-Inhibitor Deficiency. Front. Immunol..

[4] Firinu D., Costanzo G., Del Giacco S. (2020). Oxidative Stress in Hereditary Angioedema Caused by C1 Inhibitor Deficiency: An Interesting Finding That Deserves Further Studies. Pol. Arch. Intern. Med..

[5] Amarasekera A., Chang D., Schwarz P., Tan T. (2021). Vascular Endothelial Dysfunction May Be an Early Predictor of Physical Frailty and Sarcopenia: A Meta-Analysis of Available Data from Observational Studies. Exp. Gerontol..

[6] Imazio M., Brucato A., Maestroni S., Cumetti D., Belli R., Trinchero R., Adler Y. (2011). Prevalence of C-Reactive Protein Elevation and Time Course of Normalization in Acute Pericarditis: Implications for the Diagnosis, Therapy, and Prognosis of Pericarditis. Circulation.

[7] Gabay C., Kushner I. (1999). Acute-Phase Proteins and Other Systemic Responses to Inflammation. N. Engl. J. Med..

[8] Tsirpanlis G., Bagos P., Ioannou D., Bleta A., Marinou I., Lagouranis A., Chatzipanagiotou S., Nicolaou C. (2005). Serum Albumin: A Late-Reacting Negative Acute-Phase Protein in Clinically Evident Inflammation in Dialysis Patients. Nephrol. Dial. Transplant..

[9] Czúcz J., Schaffer G., Csuka D., Walentin S., Kunde J., Prohászka Z., Füst G., Farkas H., Varga L. (2012). Endothelial Cell Function in Patients with Hereditary Angioedema: Elevated Soluble E-Selectin Level during Inter-Attack Periods. J. Clin. Immunol..

[10] Lenoir C., Rollason V., Desmeules J.A., Samer C.F. (2021). Influence of Inflammation on Cytochromes P450 Activity in Adults: A Systematic Review of the Literature. Front. Pharmacol..

[11] Cappellini M.D., Motta I. (2015). Anemia in Clinical Practice—Definition and Classification: Does Hemoglobin Change with Aging?. Semin. Hematol..

[12] Sulmont-Rossé C., Van Wymelbeke-Delannoy V., Maître I. (2022). Prevalence of Undernutrition and Risk of Undernutrition in Overweight and Obese Older People. Front. Nutr..

[13] Kobayashi K., Nishida T., Sakakibara H. (2023). Factors Associated with Low Albumin in Community-Dwelling Older Adults Aged 75 Years and Above. Int. J. Environ. Res. Public Health.

[14] Lin T., Zhao Y., Xia X., Ge N., Yue J. (2020). Association between Frailty and Chronic Pain among Older Adults: A Systematic Review and Meta-Analysis. Eur. Geriatr. Med..

[15] Honda D., Li P., Jindal A., Katelaris C., Zhi Y., Thong B., Maurer M., Farkas H., Zanichelli A., Longhurst H. (2024). Uncovering the True Burden of Hereditary Angioedema Due to C1-Inhibitor Deficiency: A Focus on the Asia-Pacific Region. J. Allergy Clin. Immunol..

[16] Guan X., Sheng Y., Liu S., He M., Chen T., Zhi Y. (2024). Epidemiology, Economic, and Humanistic Burden of Hereditary Angioedema: A Systematic Review. Orphanet J. Rare Dis..

[17] Longhurst H.J., Bork K. (2019). Hereditary Angioedema: An Update on Causes, Manifestations and Treatment. Br. J. Hosp. Med..

[18] De Falco D., Misceo D., Carretta G., Gioco G., Lajolo C., Petruzzi M. (2025). Oro-Facial Angioedema: An Overview. Immuno.

[19] Demirturk M., Akpınar T., Kose M., Gelincik A., Çolakoğlu B., Buyukozturk S. (2017). Endocan: A Novel Marker of Endothelial Dysfunction in C1-Inhibitor-Deficient Hereditary Angioedema. Int. Arch. Allergy Immunol..

[20] Wu M., Bova M., Berra S., Senter R., Parolin D., Caccia S., Cicardi M., Bhatt D.L., Bhatt A.B. (2020). The Central Role of Endothelium in Hereditary Angioedema Due to C1 Inhibitor Deficiency. Int. Immunopharmacol..

[21] Ferrara A., Cristinziano L., Petraroli A., Bova M., Gigliotti M.C., Marcella S., Galdiero M.R., Granata F., Marone G., Loffredo S. (2021). Roles of Immune Cells in Hereditary Angioedema. Clin. Rev. Allergy Immunol..

[22] Salemi M., Mandalà V., Muggeo V., Misiano G., Milano S., Colonna-Romano G., Ferrara N., Mazzola G., De Leo G. (2016). Growth Factors and IL-17 in Hereditary Angioedema. Clin. Exp. Med..

[23] Allen S. (2017). Systemic Inflammation in the Genesis of Frailty and Sarcopenia: An Overview of the Preventative and Therapeutic Role of Exercise and the Potential for Drug Treatments. Geriatrics.

[24] Phansopkar P. (2021). Fear Avoidance Model of Kinesiophobia and Rehabilitation. J. Med. Pharm. Allied Sci..

[25] Wlazło M., Szlacheta P., Grajek M., Staśkiewicz-Bartecka W., Rozmiarek M., Malchrowicz-Mośko E., Kuczmarska A., Woźnica E. (2025). The Impact of Kinesiophobia on Physical Activity and Quality of Life in Patients with Chronic Diseases: A Systematic Literature Review. Appl. Sci..

[26] Hews-Girard J., Goodyear M. (2021). Psychosocial Burden of Type 1 and 2 Hereditary Angioedema: A Single-Center Canadian Cohort Study. Allergy Asthma Clin. Immunol..

[27] Maas C., López-Lera A. (2019). Hereditary Angioedema: Insights into Inflammation and Allergy. Mol. Immunol..

[28] Bork K., Hardt J., Witzke G. (2012). Fatal Laryngeal Attacks and Mortality in Hereditary Angioedema Due to C1-INH Deficiency. J. Allergy Clin. Immunol..

[29] Levy D., Nagase F., Cheung A., Manning M. (2024). Quality of Life and Burden of Disease in Patients with Hereditary Angioedema and Their Caregivers. Ann. Allergy Asthma Immunol..

[30] Nordenfelt P., Dawson S., Wahlgren C., Lindfors A., Mallbris L., Björkander J. (2014). Quantifying the Burden of Disease and Perceived Health State in Patients with Hereditary Angioedema in Sweden. Allergy Asthma Proc..

